# EMERGING TECHNOLOGY AND OLDER ADULTS: DEVELOPING A CONNECTED AND AUTONOMOUS VEHICLE TRAINING PROGRAM

**DOI:** 10.1093/geroni/igae098.1579

**Published:** 2024-12-31

**Authors:** Renée St Louis, Lisa Molnar, David Eby, Jennifer Zakrajsek, Nicole Zanier

**Affiliations:** University of Michigan Transportation Research Institute, Ann Arbor, Michigan, United States; University of Michigan, Ann Arbor, Michigan, United States; University of Michigan, Ann Arbor, Michigan, United States; University of Michigan Transportation Research Institute, Ann Arbor, Michigan, United States; University of Michigan Transportation Research Institute, Ann Arbor, Michigan, United States

## Abstract

Mobility is vital for the well-being and quality of life of older adults. Emerging technologies, such as connected and automated vehicles (CAVs), hold promise for extending safe and independent mobility for older adults, however, challenges remain in ensuring that CAVs are accessible, acceptable, affordable, and otherwise inclusive for older adults. While personal vehicles with Level 2 automation are available, the public’s exposure to more advanced levels of automation remains limited. The first phase of this research examined benefits and barriers to using CAVs using a two-phase qualitative approach consisting of four 90-minute nominal and focus group sessions. During the introduction, participants were shown a short video defining CAVs and were asked to think about vehicles with Level 3-5 automation during the discussions. Results revealed a strong desire among older adults for hands-on CAV experiences to increase perceived safety and trust in these new technologies. Based on these findings, a second phase of research involved the development of a CAV training program for older adults to be tested at Mcity, an autonomous vehicle testing facility. The training program consists of an informational overview combined with a hands-on orientation to the location and operation of various advanced vehicle technologies and automation features, using a vehicle with Level 3 automation. During the orientation, participants are shown icons associated with the technologies, and their function, capabilities, and limitations are demonstrated. Detailed findings from both phases of the research will be discussed as well as their implications for future mobility options for older adults.

